# Sequencing and *De Novo* Assembly of the Transcriptome of the Glassy-Winged Sharpshooter (*Homalodisca vitripennis*)

**DOI:** 10.1371/journal.pone.0081681

**Published:** 2013-12-10

**Authors:** Raja Sekhar Nandety, Shizuo G. Kamita, Bruce D. Hammock, Bryce W. Falk

**Affiliations:** 1 Department of Plant Pathology, University of California Davis, Davis, California, United States of America; 2 Department of Entomology and UC Davis Comprehensive Cancer Research Center, University of California Davis, Davis, California, United States of America; Queen's University Belfast, United Kingdom

## Abstract

**Background:**

The glassy-winged sharpshooter *Homalodisca vitripennis* (Hemiptera: Cicadellidae), is a xylem-feeding leafhopper and important vector of the bacterium *Xylella fastidiosa;* the causal agent of Pierce’s disease of grapevines. The functional complexity of the transcriptome of *H. vitripennis* has not been elucidated thus far. It is a necessary blueprint for an understanding of the development of *H. vitripennis* and for designing efficient biorational control strategies including those based on RNA interference.

**Results:**

Here we elucidate and explore the transcriptome of adult *H. vitripennis* using high-throughput paired end deep sequencing and *de novo* assembly. A total of 32,803,656 paired-end reads were obtained with an average transcript length of 624 nucleotides. We assembled 32.9 Mb of the transcriptome of *H. vitripennis* that spanned across 47,265 loci and 52,708 transcripts. Comparison of our non-redundant database showed that 45% of the deduced proteins of *H. vitripennis* exhibit identity (*e*-value ≤1^−5^) with known proteins. We assigned Gene Ontology (GO) terms, Kyoto Encyclopedia of Genes and Genomes (KEGG) annotations, and potential Pfam domains to each transcript isoform. In order to gain insight into the molecular basis of key regulatory genes of *H. vitripennis*, we characterized predicted proteins involved in the metabolism of juvenile hormone, and biogenesis of small RNAs (Dicer and Piwi sequences) from the transcriptomic sequences. Analysis of transposable element sequences of *H*. *vitripennis* indicated that the genome is less expanded in comparison to many other insects with approximately 1% of the transcriptome carrying transposable elements.

**Conclusions:**

Our data significantly enhance the molecular resources available for future study and control of this economically important hemipteran. This transcriptional information not only provides a more nuanced understanding of the underlying biological and physiological mechanisms that govern *H. vitripennis*, but may also lead to the identification of novel targets for biorationally designed control strategies.

## Introduction

The transcriptome is a complete set of RNA transcripts produced inside an organism at a particular time, and represents a genomic blueprint of that organism. Unlocking the complexity of the transcriptome is essential for interpreting the functional elements of a genome which can be applied for more effective downstream applications including developing genome based biorational control strategies such as those based on RNA interference. RNA-seq technology has been used for many organisms to reveal an increasing number of novel transcripts and sequence variations that result from alternate splicing and gene fusion events [1]. Similar efforts using next generation sequencing and *de novo* assembly approaches have been successfully used to decipher the transcriptome information for non-model insect species including poplar leaf beetle (*Chrysomela tremulae)* [2], orange wheat blossom midge (*Sitodiplosis mosellana*) [3], tobacco hornworm (*Manduca sexta*) [4], soybean aphid (*Aphis glycines)* [5], whitefly (*Bemicia tabaci*) [6], oriental fruit fly (*Bactrocera dorsalis*) [7], brown plant hopper (*Nilaparvata lugens*) [8], rice leaf folder (*Cnaphalocrosis medinalis*) [9], gall midge (*Mayetiola destructor*) [10], diamond black moth (*Plutella xylostella*) [11], walking stick (*Timema cristinae*) [12], blowfly (*Lucilia sericata*) [13], housefly (*Musca domestica)* [14], mountain pine beetle (*Dendroctonus ponderosae*) [15], western tarnished plant bug (*Lygus hesperus*) [16] and Asian tiger mosquito (*Aedes albopictus*) [17]. The genome size of insects can vary from less than 100 megabases to larger than 10 gigabases [18] with variation often resulting from transposon activity that inflates or deflates the genome [19].

The glassy-winged sharpshooter, *Homalodisca vitripennis* (Hemiptera: Cicadellidae), is a xylem-feeding leafhopper. *H*. *vitripennis* is a very economically important pest of a wide range of plants including *Citrus* spp., grapes (*Vitis vinifera*), and almonds (*Prunus dulcis*) [20]. This insect also serves as a very robust vector of the bacterium *Xylella fastidiosa,* the causal agent of Pierce’s disease of grapevines and citrus variegated chlorosis disease [21]. Our recent efforts have aimed at possible management of *H. vitripennis* through the use of RNAi strategies to effectively target critical host mRNAs [22], [23]. RNAi is a widely used tool to knock down and analyze the function of genes, especially in non-model organisms where the systematic recovery of mutants is not feasible. This approach, however, is limited in *H*. *vitripennis* because of the lack of information of potential targets. In a few highly established model organisms, such as *Drosophila*, hairpin constructs can be used to overexpress dsRNA in particular tissues and at specific developmental stages [24]. Similar strategies have been tested against adult *H. vitripennis* [20], [25] and other insects [26]. However, robust systemic RNAi is absent in insects [27] because they lack a functional RNA dependent RNA polymerase (RdRP) that amplifies small RNAs.

Through transcriptome sequencing of *H. vitripennis*, we sought to gain a preliminary understanding of mRNAs that might be associated with molting, metamorphosis, and development, and to identify possible RNAi targets. Insect metamorphosis can be influenced by different classes and levels of juvenile hormones (JHs); the levels of JHs in turn can be influenced by JH metabolic enzymes (juvenile hormone esterase, JHE; juvenile hormone epoxide hydrolase, JHEH; juvenile hormone acid methyl transferase, JHAMT). Similarly, developmental regulation and genome reshaping in insects is guided through the activity of retro elements, that are widely recognized to influence insect evolution and physiology [19]. Interestingly, the expression and activity of transposable elements (TEs) is now known to be epigenetically regulated by mechanisms that may include post-translational modifications of histones, DNA methylation, and production of non-coding small RNAs [28]. Transposable genetic elements (TEs) are an array of DNA fragments that have the ability to move into new sites in a genome either by a cut-and-paste mechanism (transposons) or indirectly through RNA intermediates (retro transposons) [29]. TEs, due to their autonomous replicative abilities, occupy a large portion of genomes of higher eukaryotic organisms and are present in a reversibly inactive stage [28]. TEs belonging to the TC1/mariner class [30], *Tigger* and *pogo* elements [31], *piggyBac* [32], [33] and *jockey* [34] have been previously identified from insects. Further, data from genome sequencing projects in different organisms show that TEs constitute a large portion of eukaryotic genomes [28], [29], [35]. For example, systematic analysis of the 12 sequenced genomes of *Drosophila* species revealed the variation of TE content from 2.7% to ∼25% of the host genomes, although the relative abundance of different groups of TEs is conserved across most of the species [36]. TEs constitute roughly 40% of the genome of silkworms [37], [38] and similar 47% of the genome of the mosquitoes *Aedes aegypti* [39].

Transcriptome analysis of *H. vitripennis* can provide insights into potential genomic targets and can enhance our ability to interpret critical functional pathways and the underlying molecular mechanisms of their regulation. To better understand the complexity of the *H. vitripennis* transcriptome, we performed Illumina-based sequencing of RNAs isolated from adult *H. vitripennis*. These analyses identified a substantial number of new and novel transcripts which significantly improve our understanding on the genome prints of *H. vitripennis*. Our results provide a global view of the transcriptome of *H. vitripennis* and pave the way for its further analysis.

## Materials and Methods

### Maintenance of *H. vitripennis* and RNA Extraction

A colony of *H. vitripennis* was maintained at the University of California-Davis Contained Research Facility (CRF) in cages containing a mixture of host plants as previously described [20]. Eight day-old adult insects (males and females) were collected in three different pools of ∼50 insects each. Total RNA samples for each insect group were extracted using TRIzol reagent (Invitrogen, Carlsbad, CA, USA).

### Library Preparation and Sequence Data Generation

Total RNA (4 µg per library) was used as the template to construct paired-end indexed Illumina mRNA libraries by Eureka Genomics (Hercules, CA). The quality of RNA was checked by BioAnalyzer and samples with high RIN value were processed according to Illumina’s mRNA sequencing sample preparation guide. Briefly, mRNA was enriched, and fragmented using divalent cations. The cleaved RNA fragments were copied into first strand cDNA using reverse transcriptase and random primers. Second strand cDNA synthesis was generated using DNA Polymerase I and RNaseH. These cDNA fragments were followed through an end repair process, the addition of single ‘A’ base and ligation of the adapters. The products were then purified and enriched with 18 cycles of PCR. Sequencing of the RNA libraries was performed using 51 cycle paired end reads by the Illumina Genome Analyzer. The mRNA raw sequence data was deposited to the SRA database at NCBI with Biosample accession number SAMN02319001. SRA experiments depositions can be accessed via reference numbers SRX336675 and SRR954044. The data can also be accessed through a BioProject accession number PRJNA215794.

### Sequence Analysis

Prior to analysis, adapter nucleotides were trimmed and the sequences were *de novo* assembled using the latest version of Velvet assembler, coupled with the Oases [40] package. The Oases package operates on the output of the Velvet assembler, utilizing the pairing information in the sequencing reads to identify and group transcript isoforms into appropriate loci. Homologous protein domains from translated transcriptomic sequences of *H*. *vitripennis* were identified by searching against the Pfam database [41] using HMMER [42]. BLAST2GO [43], [44] was used to assign putative functionalities, GO terms, and KEGG (Kyoto Encyclopedia of Genes and Genomes) based metabolic pathways [26], [45]. Final GO term assignments were defined based on a 10% filter for all three processes profiled at level 2. All other settings for the analysis were maintained at their defaults (Table S3). For phylogenetic analysis, putative vacuolar ATPase (vATPase), juvenile hormone acid methyl transferase (JHAMT), juvenile hormone epoxide hydrolase (JHEH), Dicer (DCR), and Piwi, protein sequences of *H. vitripennis* and other insect species were aligned using the ClustalW algorithm [46] and analyzed in MEGA 5 [47] using default maximum parsimony settings with 500 bootstrap replications.

### Validation of Gene Expression

Reverse transcription PCR (RT-PCR) was performed to validate the transcriptomic information using the total RNA from three individual adult glassy-winged sharpshooter insects. Psyllid adults (*Diaphorina citri*) were used as negative controls. One microgram of total RNA was reverse transcribed into single-stranded cDNA using the Bio-Rad iScript Reverse Transcription Kit. The ubiquitin gene from *H. vitripennis* was used as an internal control. The gene expression was checked for the following *H. vitripennis* transcripts; *actin*, *cuticle*, *chitin deacetylase*, *juvenile hormone esterase* (*jhe*), *juvenile hormone epoxide hydrolase* (*jheh*), *juvenile hormone acid methyl transferase* (*jhamt*), *serpin b6*, *sugar transporter*, *vacuolar ATPase*, *dicer*, *argonaute-2* and *zinc metalloproteinase*. The PCR primer sequences used for the amplification of the *H.vitripennis* sequences are included as supplemental information (Table S1). The biological activity of the JHEH protein encoded by transcript #8298 of *H*. *vitripennis* was biologically characterized as recently described [48]. Briefly, a cDNA corresponding to transcript number 8298 was PCR-amplified from a single 5^th^ instar nymph. A recombinant baculovirus, AcHovimEH1, expressing this cDNA was generated and AcHovimEH1 was used to express recombinant protein, Hovi-mEH1, in High Five cells. A microsomal preparation of Hovi-mEH1 was then made from AcHoviEH1-infected High Five cells. Partition assays with three general epoxide containing substrates (*cis*-stilbene oxide, *trans*-stilbene oxide, and *trans*-diphenylpropene oxide) and JH III were then performed to characterize the specific activity of Hovi-mEH1 [49].

## Results

### Transcriptome Assembly

**Figure 1:**
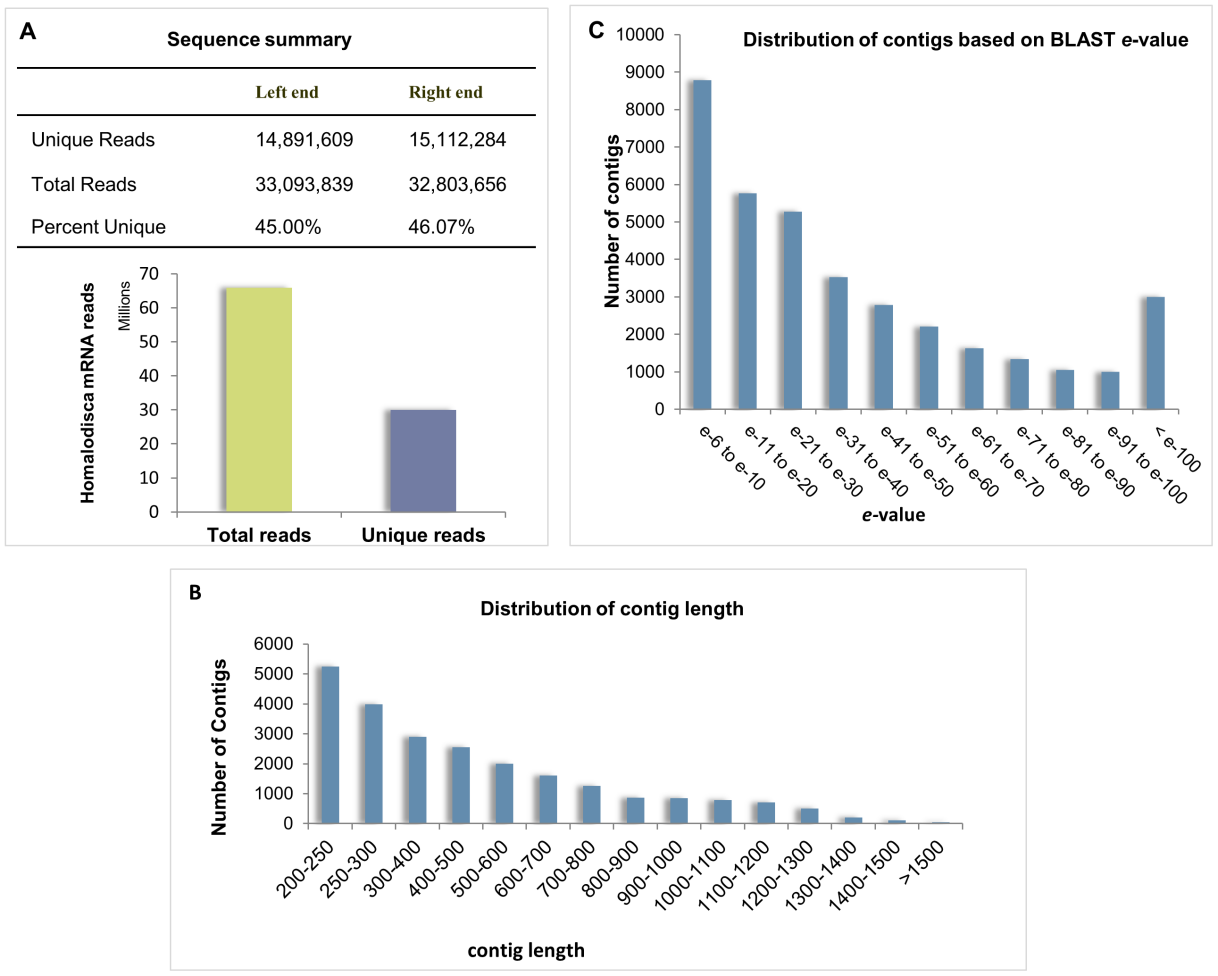
Sequence summary of the transcriptome of *H. vitripennis*. **A**) Summary of mRNAseq library sequences. Total and unique reads from both sequencing directions are shown. The reads, represented in millions, are shown along the Y-axis while the representative classes of reads are shown along the X-axis. **B**) Contig length distribution is shown as a bar chart. The contig length is represented along the X-axis while the number of contigs is shown along the Y-axis. **C**) Contig distribution based on their BLAST *e*-values is shown as a bar chart. The *e*-value is represented along the X-axis while the number of contigs is shown along the Y-axis.

In order to obtain a global snapshot view of the transcriptome of *H. vitripennis*, high-throughput deep sequencing experiments were performed using Illumina mRNA sequencing technology with poly (A)-enriched RNAs from pooled samples of adult *H. vitripennis*. After removal of low quality reads, a total of 32,803,656 paired-end reads were obtained. Approximately 45% of the sequence reads were found to be unique to the *H. vitripennis* genome (Figure 1A). All of the resulting paired-end sequence reads were assembled into 52,708 transcripts across 47,265 loci. The average transcript length was 624 nucleotides, with the minimum and maximum lengths of 200 and 14,493 nucleotides respectively (Figure 1B). The N50 value of 820 tells us that majority of the transcripts are over 800 nucleotides in a manner that was consistent with the average length obtained. We were able to assemble approximately 32.9 Mb of the *H. vitripennis* transcriptome from our sequencing study. The contigs were further sorted based on their length as represented in Figure 1B. A high number of BLAST hits (8,786) showed a BLAST *e*-value range between *e* ^−6^ to *e* ^−10^ indicating a high degree of confidence in the contigs generated (Figure 1C).

### Comparative Analysis and Homology Distribution

**Figure 2:**
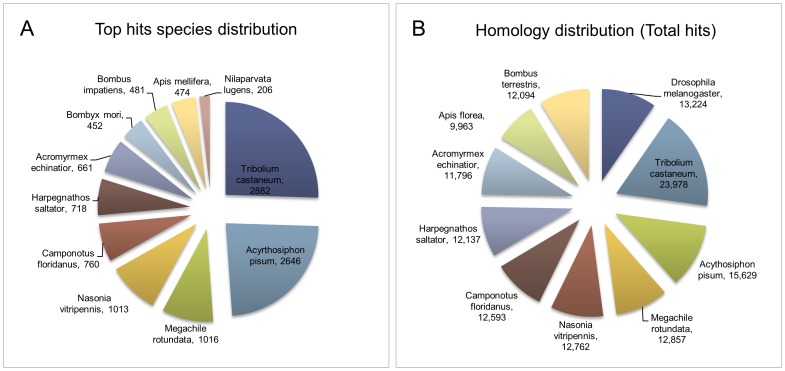
BLAST distribution and homology of the *H. vitripennis* contigs. Pie diagrams showing the homology distribution **A**) Top hit species distribution of *H. vitripennis* contigs against those of other insect species and **B**) Total species homology distribution of *H. vitripennis* contigs against other insects.

Comparative analyses of *H. vitripennis* transcripts with those from other insects were performed using TBLASTX analysis against non-redundant (nr) databases (Table S2). A TBLASTX analysis cutoff *e*-value of ≤1*e* ^−5^ was used to compare the *H. vitripennis* deduced proteins. Approximately 45% (23,547) of the *H. vitripennis* deduced proteins were found to be identical to proteins in non-redundant insect databases (Figure 2A and ). The remaining proteins generated an *e*-value higher than the cutoff, suggesting that they might be either novel proteins or from other cellular organisms (e.g. microbes) that are associated with *H. vitripennis*. Alternatively, these sequences could also correspond to the untranslated regions of *H. vitripennis* genome. The identity of (45%) *H. vitripennis* proteins to known proteins in nr databases is consistent with that reported for brown planthopper (*N. lugens*) (56%) [50], pine shoot beetle (*Tomicus yunnanensis*) (54%) [51], and western tarnished plant bug (55%) [16]; and is considerably higher than that reported for other non-model insect pests [52].

The top homology hits identified by TBLASTX analysis were from the insects *T. castaneum* (26%), *Acyrthosiphon pisum* (25%), *Megachile rotundata* (9%), *Nasonia vitripennis* (9%), *Camponotus floridanus* (7%) *Harpegnathos saltator* (6%), *Acromyrex echinator* (6%), *Apis mellifera* (4%), *N. lugens* (2%), and *Bombus impatiens* (5%) (Figure 2A). The large number of sequence hits between *H. vitripennis* (Hemiptera) and *T. castaneum* (Coleptera) was not entirely unexpected given the enormity of the available sequence data from *Tribolium* and completeness of the sequence annotation in this species. The 24% sequence homology between the *H. vitripennis* transcriptome and that of the pea aphid, *A. pisum* (Hemiptera), is in accordance with their phylogenetic relatedness. We expect that the number of homologous sequences between *H. vitripennis* and *A. pisum* will increase as annotations of both sequences are completed. Interestingly, sequences from the genome of *N. vitripennis*, a predatory wasp of *H. vitripennis*, showed approximately 9% homology to that of *H. vitripennis* suggesting that there might be some host specificity recognition.

The presence of *H. vitripennis* homologous sequences in other insect orders was examined in order to identify conserved sequences (Figure 2B). Among the total hits of the species distribution, conservation of the protein sequences among *A. pisum*, *T. castaneum*, *Drosophila melanogaster*, *M. rotundata*, *B. terrestris*, *A. mellifera* and *Anophles gambiae* was observed. This list is only a partial list of the total species shown in Figure 2B.

#### Functional classification of *H. vitripennis* transcriptome

**Figure 3:**
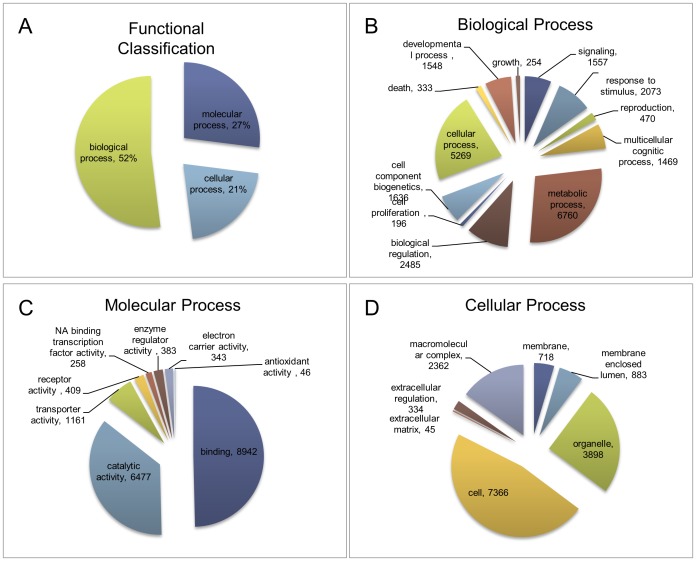
Functional distribution of deduced proteins of the *H. vitripennis* transcriptome. Pie diagrams showing **A**) Functional distribution of deduced proteins **B**) Biological classification of deduced proteins **C**) Molecular distribution of deduced proteins and **D**) Cellular distribution of deduced *H. vitripennis* proteins.

The whole insect transcriptome of *H.vitripennis* was classified into three main functional processes: biological, molecular, and cellular. Approximately half (52%) of the functional proteins of *H*. *vitripennis* were classified in the biological process category (Figure 3A). The remaining functional proteins were classified into the molecular processes category (27%) and cellular processes category (21%). The distribution of GO terms within the ontology categories was consistent with other insect transcriptomes [16].

The biological processes category proteins were further classified into the sub-classifications: metabolic processes (6,760 proteins), multicellular cognitic processes (1,469 proteins), cell component biogenesis (1,636 proteins), cellular processes (5,269 proteins), developmental processes (1,548 proteins), growth (254 proteins), biological regulation (2,845 proteins), and signaling processes (1,557 proteins) (Figure 3B). The *H. vitripennis* transcriptome profile displayed a strong repertoire of the proteins (6,760 proteins) that were predicted to be involved in metabolic processes.

Molecular function distributions from the Gene Ontology (GO) analyses showed that in *H. vitripennis*, the expressed genes were mostly linked to molecular binding activity (8,942 proteins) or categorized as catalysts (e.g., hydrolase and oxidoreductase: 6,477 proteins) (Figure 3C). The other major groups of proteins under molecular processes are involved with enzyme regulation (383 proteins), transporter activity (1,161 proteins), and receptor related functions (409 proteins) (Figure 3C). The contigs/singletons involved in electron carrier activity and NA binding transcription factors constitute the next most abundant categories amounting to 343 and 258 proteins, respectively (Figure 3C).

Among the cellular processes (Figure 3D), the majority of the *H. vitripennis* proteins were found to be involved in cell function (∼47%) or were organellar proteins (25%). The other major cellular processes proteins were membrane associated proteins or proteins (15%) involved with the formation of macromolecular complexes (Figure 3D).

### Metabolic Pathways of *H. vitripennis*

**Figure 4:**
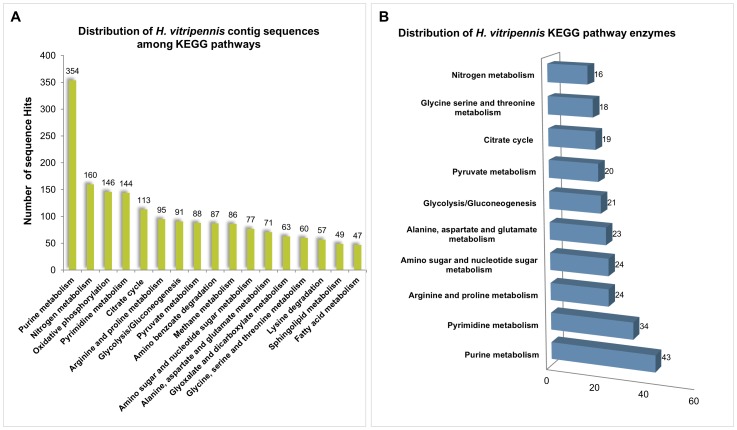
Summary of *H. vitripennis* KEGG pathways. **A**) Distribution of KEGG pathway sequences of *H*. *vitripennis* is shown as a bar chart. The number of sequence hits is shown along the Y-axis while the different KEGG pathways are shown along the X-axis. **B**) The top ten KEGG pathway distributions in the *H. vitripennis* genome are represented as a bar diagram. KEGG pathway enzymes were represented along Y-axis and the numbers of enzymes are represented along X-axis.

The Kyoto Encyclopedia of Genes and Genomes (KEGG) database [26], [45] was used to identify potential pathways represented in the *H. vitripennis* transcriptome. Based on KEGG pathway mapping analysis and annotation, we were able to map a total of 1,788 *H. vitripennis* sequences to the 17 KEGG pathway maps for biological interpretation of higher level systemic functions (Figure 4A). *H. vitripennis* KEGG classification analysis identified 354 transcripts that were potentially involved in purine metabolism followed by 160 and 146 transcripts potentially involved in nitrogen metabolism and oxidative phosphorylation, respectively (Figure 4A). Further, KEGG metabolic pathways presented in the current dataset include Nucleotide Metabolism (498 transcripts), Protein Metabolism (303), Lipid Metabolism (97), and Carbohydrate Metabolism (259) (Figure 4A). A number of genes were found to be upregulated in energy metabolism pathways is consistent with the higher rate of metabolism and higher activity of insects. Consistent with the identification of the sequences that mapped to the KEGG pathways, enzymatic distribution follows a similar pattern (Figure 4B). The distribution of KEGG pathway enzymes in *H. vitripennis* was similar to that found in western tarnished plant bug [16].

### Identification of Putative Proteins Involved in Small RNA Biogenesis and Other Proteins of Specific Interest

**Figure 5:**
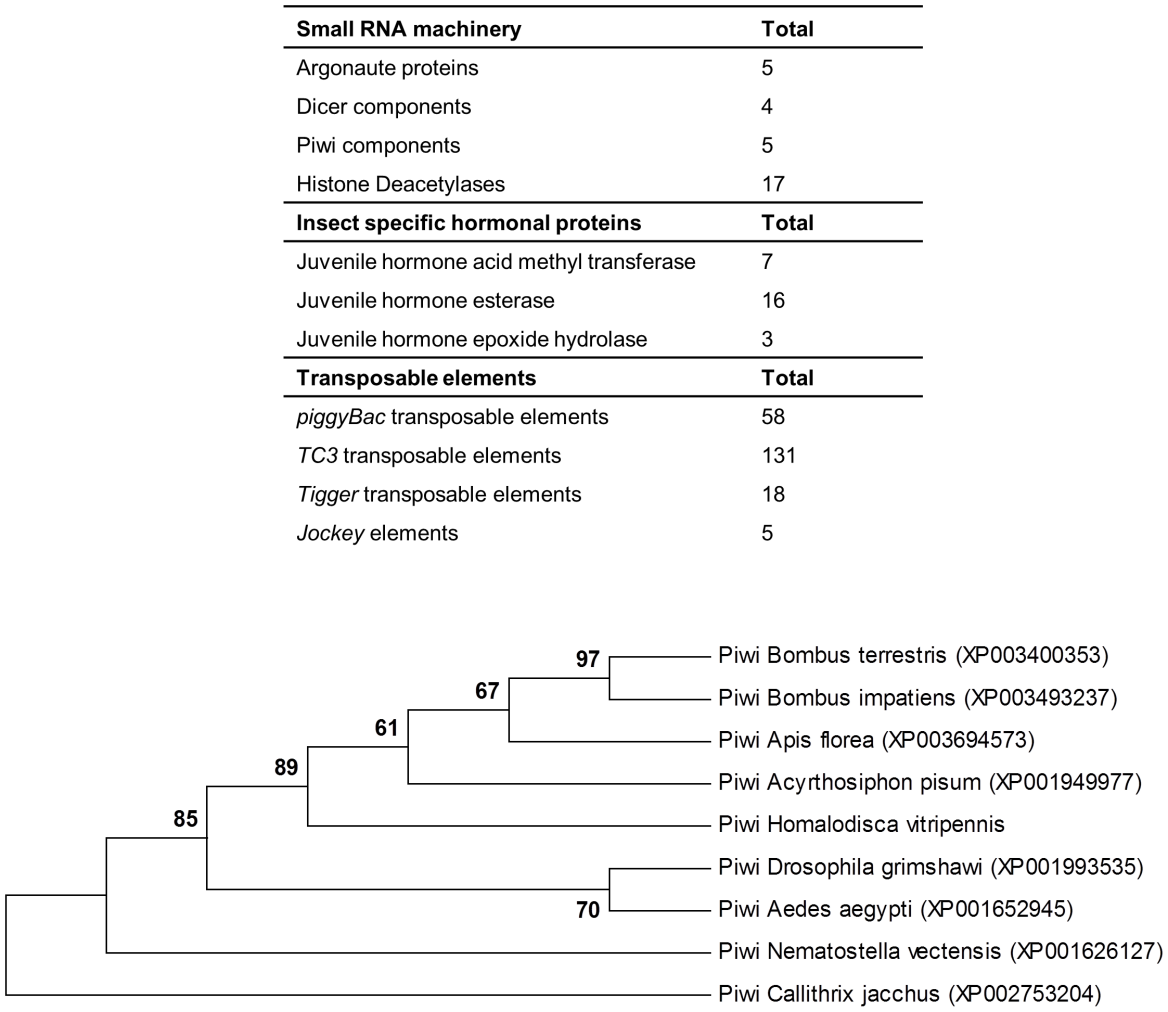
Distribution and phylogeny of deduced *H. vitripennis* Piwi proteins. Upper panel. Table showing the distribution of proteins involved in small RNA biogenesis and epigenetic processes, juvenile hormone metabolism, and genome plasticity. **Bottom panel.** The evolutionary relatedness of Piwi proteins of *H. vitripennis* with other insect Piwi proteins is shown. The evolutionary history was inferred using the Neighbor-Joining method. The percentage of replicate trees in which the associated taxa clustered together in the bootstrap test (500 replicates) is shown next to the branches.

**Figure 6:**
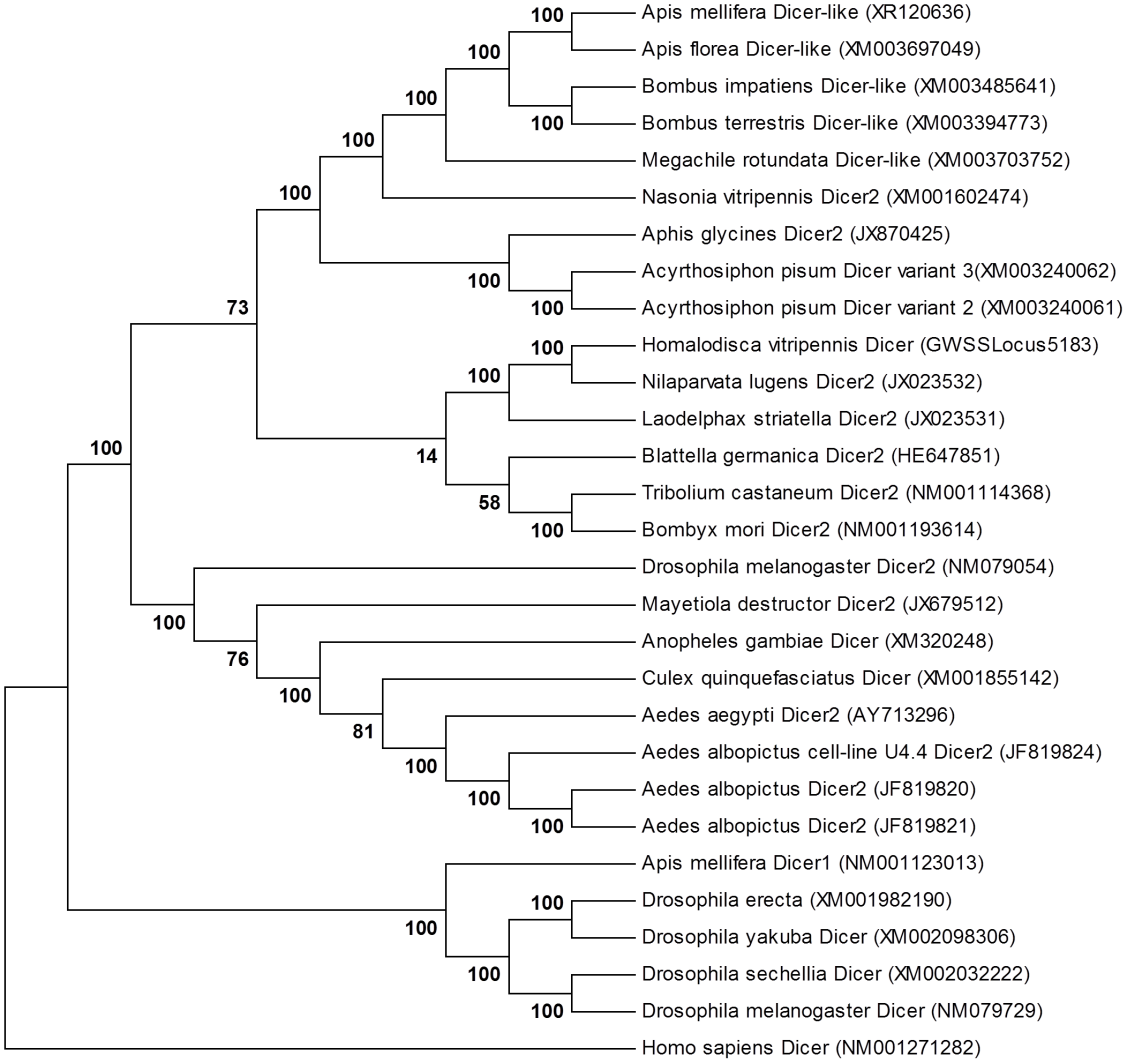
Distribution and phylogeny of deduced *H. vitripennis* Dicer proteins. The evolutionary relationship of *H. vitripennis* Dicer protein with other insect Dicer proteins is shown. The bootstrap consensus tree inferred from 500 replicates is taken to represent the evolutionary history of the taxa analyzed. Branches corresponding to partitions reproduced in less than 50% bootstrap replicates are collapsed.

RNA interference (RNAi) pathways are important modulators of gene expression. RNAi operates by degrading RNA target molecules through the interaction of short (21–30 nucleotide) RNAs [26]. RNAi components have been reported to have a role in the nucleus, as they are involved in epigenetic regulation and heterochromatin formation [18]. It was recently shown that key RNAi components Dicer 2 (DCR2) and Argonaute 2 (AGO2) associate with chromatin (with a strong preference for euchromatic, transcriptionally active, loci) and interact with the core transcription machinery [18]. In our efforts to understand the epigenetic regulation of *H. vitripennis,* sequences corresponding to Dicer, Argonaute, and Piwi proteins were identified (Figure 5, upper panel). The Piwi class of *H. vitripennis* proteins showed the highest identity to Piwi proteins of *A. pisum* (89%) and some other insects (Figure 5, bottom panel). Piwi from *Callithrix jacchus* was found to be phylogenetically distant to Piwi of *H. vitripennis* (5, bottom panel). Similarly, *H. vitripennis* Dicer proteins displayed the highest identity to Dicer proteins of *A. pisum* and other hemipterans (*Myzus persicae*) (Figure 6). Histone deacetylases (HDAC’s) remove acetyl groups on histones allowing them to tightly wrap around DNA resulting in transcriptional repression. The transcriptome of *H. vitripennis* appeared to encode 17 HDAC transcripts, suggesting a fully functional epigenetic regulatory pathway (Figure 5, upper panel). It was previously reported that an amplification signal for small RNAs is lacking in hemipterans and other higher metazoans [53]. Consistent with this observation, *H*. *vitripennis* appeared to not encode an RNA dependent RNA polymerase (RdRP). It remains to be seen if an alternate mechanism plays a role in RNA amplification in metazoans as we previously reported strong small RNA profiles in adult *H. vitripennis* [54]. With so much epigenetic regulation and interlinked small RNA machinery being active, we next asked if the genome is able to maintain its genome plasticity.

The transcriptome of *H*. *vitripennis* was analyzed for the presence of transposable elements (TEs) as an indicator of genetic novelty and genome evolution [55]. From previous reports, TEs are known to occupy a substantial portion of eukaryotic genomes and play evolutionarily unique roles in maintaining diversity, integrity, and stability of genomes [35]. Among insect genomes, the repeat content varies greatly: 1% in *A. mellifera* [56] *;* 16% in *An. gambiae* [57] *;* 33% in *T. castaneum* [58], and 47% in *Aedes aegypti* [39]. There is considerable diversity, however, even when a comparison is made between genomes from insects within the same genus. The 12 sequenced genomes of *Drosophila* species, for example, have repeat content that vary from 2.7% to 25% [36]. Previous reports from comparative genome sequencing of three closely related parasitoid wasps, *Nasonia vitripennis*, *N. giraulti*, and *N. longicornis* showed that the TE diversity is 30% higher in *Nasonia* (2.9 TE types/Mb) than *Bombyx mori* (2.1 TE types/Mb), and 10-fold higher than that found in the average dipteran [59]. The TE diversity (3.7% vs. 16%) exists even among the coleopterans *Tribolium castaneum* [58] and *Diabrotica virgifera* [60]. Analysis of the transcriptome *of H*. *vitripennis* identified four different types of TEs based on the BLAST homology searches that represented approximately 1% of the transcriptome (Figure 5). We identified 131, 18, 5 and 58 copies of TEs belonging to the TC3 type transposons (*TC1*/*mariner* superfamily), *Tigger* elements (*TC1*/*mariner* superfamily), *jockey* elements (Long Interspersed Elements (LINEs) that replicate through copy and paste mechanism) and *piggyBac* elements, respectively. The lower percentage of TEs found in *H. vitripennis* transcriptome is in accordance with that found in *A. mellifera* genome and confirms the highly variable state of invasion by TEs in some insects.

### Identification of Gut-specific and Juvenile Hormone Metabolic Sequences of *H. vitripennis*

Our laboratory has recently shown that the effects of RNAi are more effective against gut specific genes of the potato/tomato psyllid [26]. Insect hormonal targets such as juvenile hormone acid methyl transferase (JHAMT) also appear to be effective targets of the RNAi pathway as shown in *Helicoverpa armigera* [55] and *T. castaneum* [45]. In consideration of our larger interest of developing genome-based biorational control strategies, including those based on RNAi, we sought to identify insect specific hormonal proteins and gut specific genes such as *vacuolar ATPase* (*vATPase*). Insect bioassays have clearly showed that the vATPase genes is an effective target of dsRNA-based RNAi in a broad range of insects [61]. The *vATPase* (transcript # 1161) of *H. vitripennis* was phylogenetically most closely related to *vATPase* of *A. pisum* and other insect *vATPase* sequences (Figure S1).

**Figure 7:**
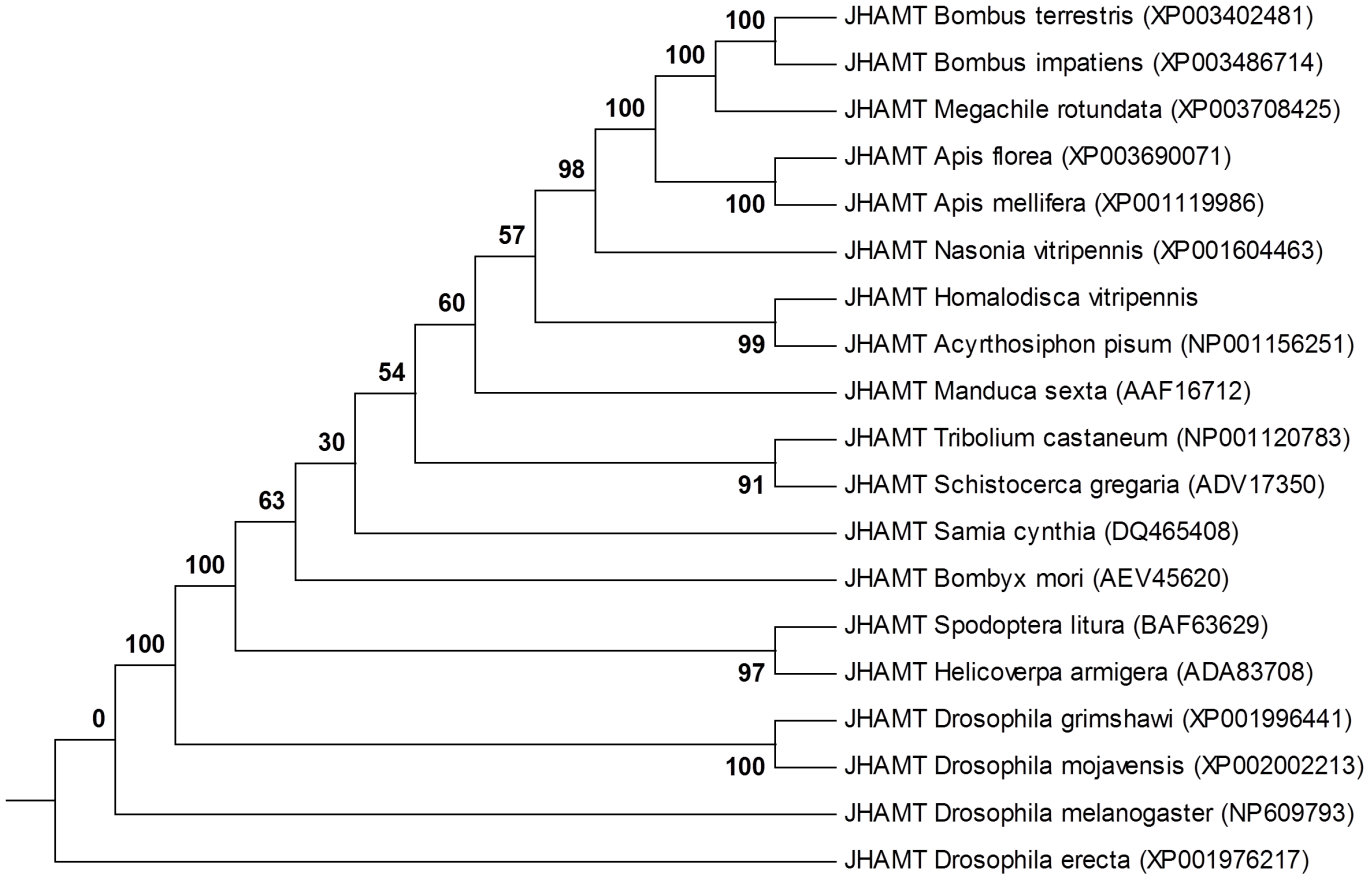
Phylogenic comparisons of juvenile hormone acid methyl transferase protein sequences of *H. vitripennis* and those other insect orders. The evolutionary relatedness of juvenile hormone acid methyl transferase (JHAMT) of *H*. *vitripennis* with other insect JHAMT proteins is shown. The evolutionary history was inferred using the Neighbor-Joining method. The bootstrap consensus tree inferred from 500 replicates is taken to represent the evolutionary history of the taxa analyzed. The right panel in the figure shows the RT-PCR gel picture validating the JHAMT transcript from *H. vitripennis* insects.

**Figure 8:**
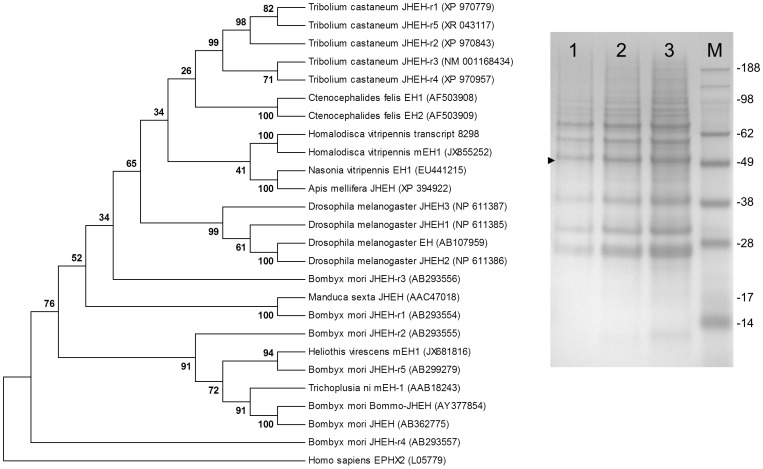
Phylogenic comparisons of juvenile hormone epoxide hydrolase protein sequences of *H. vitripennis* and those other insect orders. The evolutionary relationship of juvenile hormone epoxide hydrolase (JHEH) of *H. vitripennis* and JHEH sequences from other insects. Transcript #8298 of *H*. *vitripennis* was transiently expressed as a recombinant protein using a baculovirus expression vector and biologically characterized (see Figure 9). SDS-PAGE analysis of microsomes prepared from insect High Five cells infected with the recombinant baculovirus: lane 1, 2.1 µg; lane 2, 4.2 µg; lane 3, 8.4 µg, and lane M, protein size markers (SeeBlue Plus2, Invitrogen). The size of the markers is shown to the right in kDa. The arrowhead indicates the migration of the recombinant 52.1 kDa protein.

**Figure 9:**
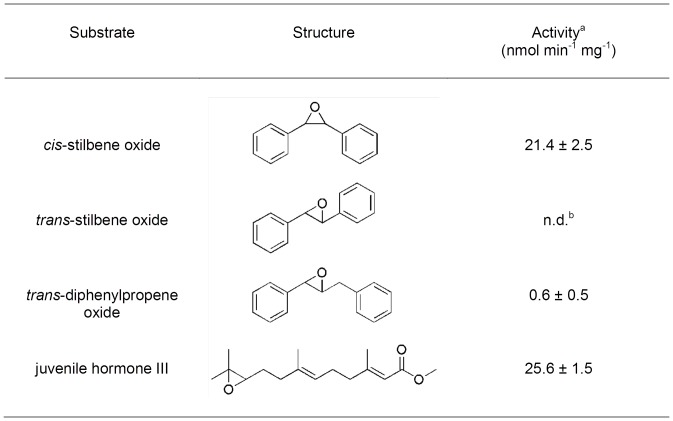
Activity of recombinant Hovi-mEH1 with various epoxide-containing substrates. The enzyme assays were performed at 30°C in buffer (100 mM sodium phosphate, pH 8.0) containing 50 µM epoxide-containing substrates, 1% (v:v) ethanol, and 0.1 mg ml^−1^ BSA. The juvenile hormone III assays also contained 10 µM 3-Octylthio-1,1,1-trifluoropropan-2-one (OTFP) and additional ethanol (2% v:v). The values shown are the mean ± standard deviation of at least three separate experiments. Similar values were obtained for these substrates with Hovi-mEH1 from another baculovirus expression construct. Activity was not detected at an assay detection limit of 0.1 nmol min^−1^ mg^−1^.

Three types of juvenile hormone (JH) metabolic gene sequences: juvenile hormone acid methyl transferase (JHAMT), juvenile hormone esterase (JHE), and juvenile hormone epoxide hydrolase (JHEH) were identified in the *H. vitripennis* transcriptome. Three, 7, and 16 isoforms of JHEH, JHAMT, and JHE, respectively, were identified in the transcriptome of *H. vitripennis* (Figure 5, upper panel). Phylogenetic analysis indicated that the *H. vitripennis* JHAMT protein is 99% identical to the *A. pisum* JHAMT protein. The JHAMT proteins from the *Drosophila* species were phylogenetically distant from putative JHAMT of *H. vitripennis* (Figure 7). Following phylogenetic analysis (Figure 8), only one of the three JHEH deduced proteins (i.e., transcript #8298) clustered with known biologically active JHEHs. Thus, transcript #8298 appeared to most likely encode a biologically functional JHEH enzyme. In order to test this hypothesis, a full-length cDNA of this isoform was cloned from fifth instar *H*. *vitripennis*. A recombinant protein, Hovi-mEH1, was expressed from this cDNA (Figure 8), and the biological activity of Hovi-mEH1 for several epoxide containing substrates including JH III was tested (Figure 9 and [48]). Hovi-mEH1 hydrolyzed both JH (25.6 nmol min^−1^ mg^−1^) and *cis*-stilbene oxide (21.4 nmol min^−1^ mg^−1^) with high specific activity (Figure 9).

### Validation of *H. vitripennis* Transcripts by RT-PCR

**Figure 10:**
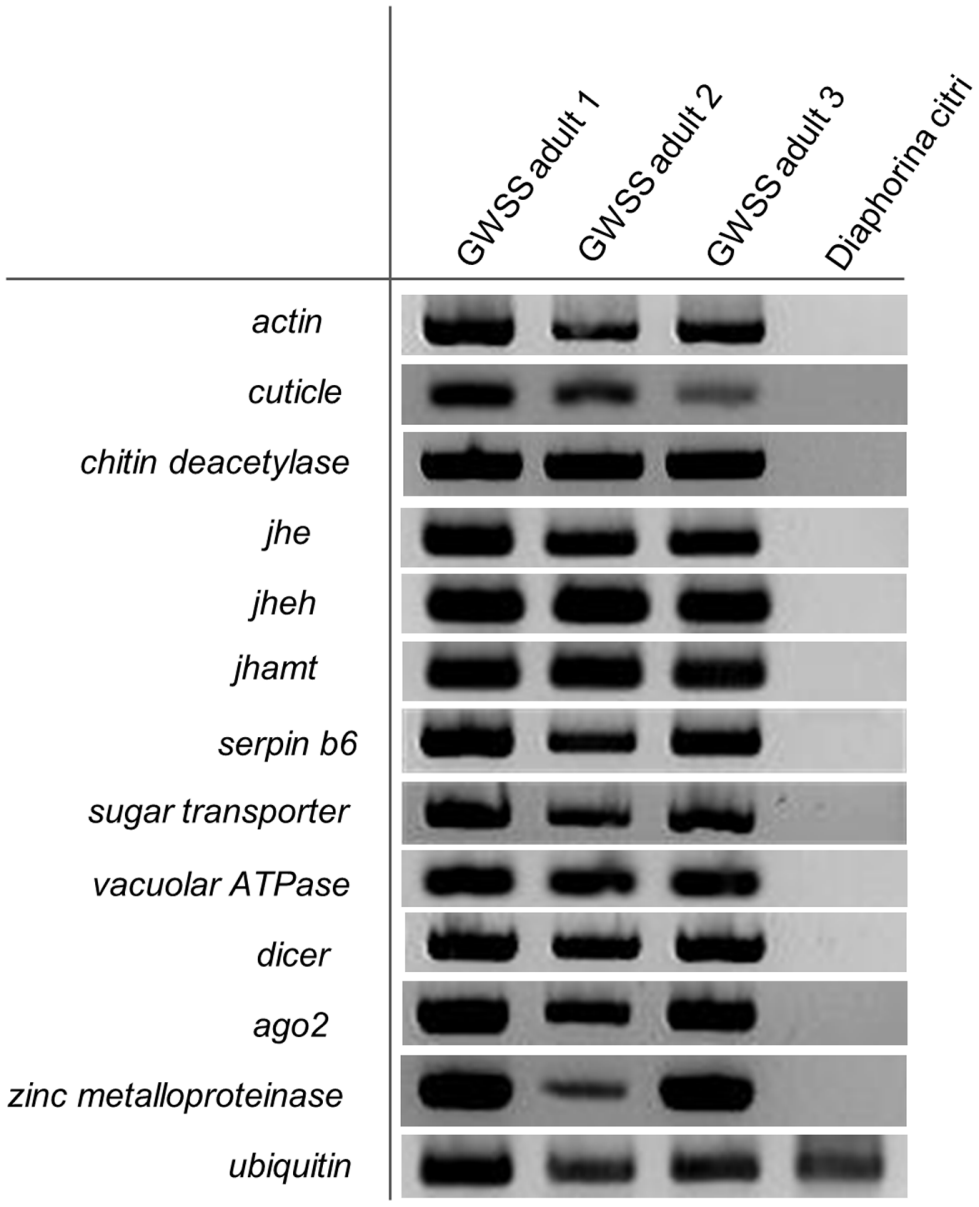
RT-PCR validation of H.vitripennis transcripts. The figure shows the RT-PCR gel picture validating the transcripts of *actin*, *cuticle*, *chitin deacetilase*, *juvenile hormone esterase* (*jhe*), *juvenile hormone epoxide hydrolase* (*jheh*), *juvenile hormone acid methyl transferase* (*jhamt*), *serpin b6*, Sugar transporter, *Vacuolar ATPase*, *Dicer*, *argonaute-2* and *Zinc metalloproteinase* from *H. vitripennis* insects. Psyllid adults (*Diaphorina citri*) insects were used as negative controls.

Reverse transcription PCR (RT-PCR) was performed to validate the transcriptomic information using the total RNA from three individual adult *H.vitripennis* insects. Psyllid adult (*Diaphorina citri*) insects were used as negative controls. One microgram of total RNA was reverse transcribed into single-stranded cDNA using the Bio-Rad iScript Reverse Transcription Kit. The ubiquitin gene from *H. vitripennis* was used as an internal control. The gene expression was checked for the following *H. vitripennis* transcripts; *actin*, *cuticle*, *chitin deacetylase*, *juvenile hormone esterase* (JHE), *juvenile hormone epoxide hydrolase* (JHEH), *juvenile hormone acid methyl transferase* (JHAMT), *serpin b6*, sugar transporter, *Vacuolar ATPase*, *Dicer*, *Argonaute-2* and *Zinc metalloproteinase* (Figure 10). Validation experiments indicate that all of the thirteen different transcripts were expressed in adult *H.vitripennis* (Figure 10).

## Discussion

Although a major pest and important vector of *X. fastidiosa*, the genetics and molecular biology of *H. vitripennis* have not been well studied. The analysis of transcriptomic sequences can provide insights into functional genomics by identifying potential biological pathways and molecular mechanisms. In this study, we applied Illumina mRNA-seq technology to partially unravel the transcriptome of *H. vitripennis.* In order to minimize variation, the *H. vitripennis* transcriptome was generated from three different pools of 50 eight day old adults. We assembled 36 Mb of sequence that covered 47,265 loci and 52,708 transcripts (Figure 1A). These data are a dramatic increase in comparison to EST data present in NCBI. Furthermore, we were able to validate all of the dataset protein sequences from *H. vitripennis* (27 listed proteins) that are present in NCBI.

Proteins involved in the RNAi pathway are found in the nucleus and cytoplasm, as they are involved in epigenetic regulation and heterochromatin formation. It was recently shown that key RNAi components Dicer 2 (DCR2) and Argonaute 2 (AGO2) associate with chromatin (with a strong preference for euchromatic, transcriptionally active, loci) and interact with the core transcriptional machinery [18]. We were able to identify the small RNA biogenesis components of *H. vitripennis* (Dicer, Argonaute, and Piwi) (Figure 5, upper panel). These sequences were found to be phylogenetically close to those of *A. pisum (* Figure 5 and Figure 6 *)*. Recently, DCR2 and AGO2 were shown to control heterochromatin formation in the nucleus, as well as repeat-induced gene silencing and transposable element mobilization [62], [63]. Recent evidence suggests for the global association of RNAi components with chromatin and their role in transcriptional regulation. Furthermore, loss of function of DCR2 or AGO2 in *Drosophila* is associated with transcriptional defects that are accompanied by the perturbation of RNA polymerase II positioning on promoters [18]. In this study we identified deacetylases that are important for the deacetylation of histones leading to transcriptional quiescence. As reported for other metazoans, we were not able to identify an RNA dependent RNA polymerase (RdRP) in *H. vitripennis* although there is evidence for a strong small RNA profile in *H. vitripennis* [54]. Systemic RNAi in plants is based on the RdRP and the spread of siRNAs through the plasmodesmata. RdRP orthologs are present in some nematodes, but they were reported to be largely absent in insects [27]. The absence of dsRNA amplification and RdRP in insects suggests that gene knockdown effects exhibited by feeding dsRNA to insects would be temporary [27]. Thus, RNAi effects achieved in the gut would likely require a continuous input of dsRNA to persist. There may, however, be alternate mechanisms for the silencing signals to be amplified or transported in the absence of a true RdRP.

Our transcriptome analysis indicated that a fully functional epigenetic regulatory protein complex is present in *H. vitripennis*. Thus, we sought to understand the genome plasticity of *H. vitripennis* through an analysis of the abundance of transposable elements. We identified four different (TEs) in the genome of *H. vitripennis*. These sequences represented approximately 1% of the genomic sequence, a level similar to that of *Apis mellifera* genome [56]. Previously *piggyBac* elements have been identified from fungi, plants, insects, crustaceans, urochordates, amphibians, fishes and mammals [33]. The *piggyBac* transposable element is currently the vector of choice for transgenesis, enhancer trapping, gene discovery and gene function studies [32], [64].The presence of *piggyBac* elements in *H. vitripennis* is highly encouraging as the elements can be used for reverse genetic studies. This relatively low abundance of TEs suggested that the genome of *H. vitripennis* was less expanded. The availability of the whole genome of *H. vitripennis* in the future might help in accurately determining the constitution of the transposable elements. In this study, we were able to further elucidate the roles of *H. vitripennis* genes in the KEGG enzymatic pathways and were able to identify gut specific genes (*vATPase*) and genes involved in the metabolism of juvenile hormone. Furthermore, we were able to validate the expression of numerous genes through RT-PCR and were able to confirm the enzymatic activity of JHEH (Figures 8–10). In summary, we performed a comprehensive analysis of the *H. vitripennis* transcriptomic data and we remain encouraged that these data will provide important leads for further study of development, sex differentiation, migratory flight, olfactory behavior, and insecticide resistance.

### Conclusions

Here, we report the elucidation of the transcriptome of adult *H. vitripennis.* Our data significantly enhance the molecular resources available for future study and control of this economically important hemipteran. This transcriptional information not only provides a more nuanced understanding of the underlying biological and physiological mechanisms that govern *H. vitripennis*, but may also lead to the identification of novel targets for biorationally designed control strategies. Among the key transcripts that we identified were those involved in juvenile hormone degradation, transposable elements, and small RNA components that are potentially crucial mediators of the architecture of the genome of *H. vitripennis.* An increased understanding of how epigenetics and small RNAs are involved in developmental regulation is essential for elucidating potential RNAi-based efforts to control *H*. *vitripennis* and related hemipterans.

## Supporting Information

Figure S1 **Phylogenetic tree of vacuolar ATPase encoded in the** ***Homalodisca vitripennis*** **transcriptome.** The evolutionary relationship of *H. vitripennis* vacuolar ATPase with other insect vATPase proteins was shown. The evolutionary history was inferred using the Neighbor-Joining method. The percentage of replicate trees in which the associated taxa clustered together in the bootstrap test (500 replicates) is shown next to the branches. The evolutionary distances were computed using the Maximum Composite Likelihood method and are in the units of the number of base substitutions per site. All ambiguous positions were removed for each sequence pair. Evolutionary analyses were conducted in MEGA5.
(TIF)

Table S1 **Primer pairs designed for the amplification of transcripts from** ***Homalodisca vitripennis*** **whole adults through the use of reverse transcriptase PCR.**
(DOCX)

Table S2 **Table containing top BLAST hits.**The table here contains a list of top blast hits found for the contigs of *H. vitripennis* transcriptome using a TBLASTX homology.
(XLSX)

Table S3 **Table summary containing Interpro-scan results.** The table here contains the summary results of Interpro-scan results for the contigs of *H. vitripennis* transcriptome.
(XLSX)
