# Supplementary figures and images for: Sequencing and De Novo Assembly of the Transcriptome of the Glassy-Winged Sharpshooter (Homalodisca vitripennis)
Source: PLoS One. 2013 Dec 10;8(12):e81681. doi: 10.1371/journal.pone.0081681 (PMC3858241; doi:10.1371/journal.pone.0081681)

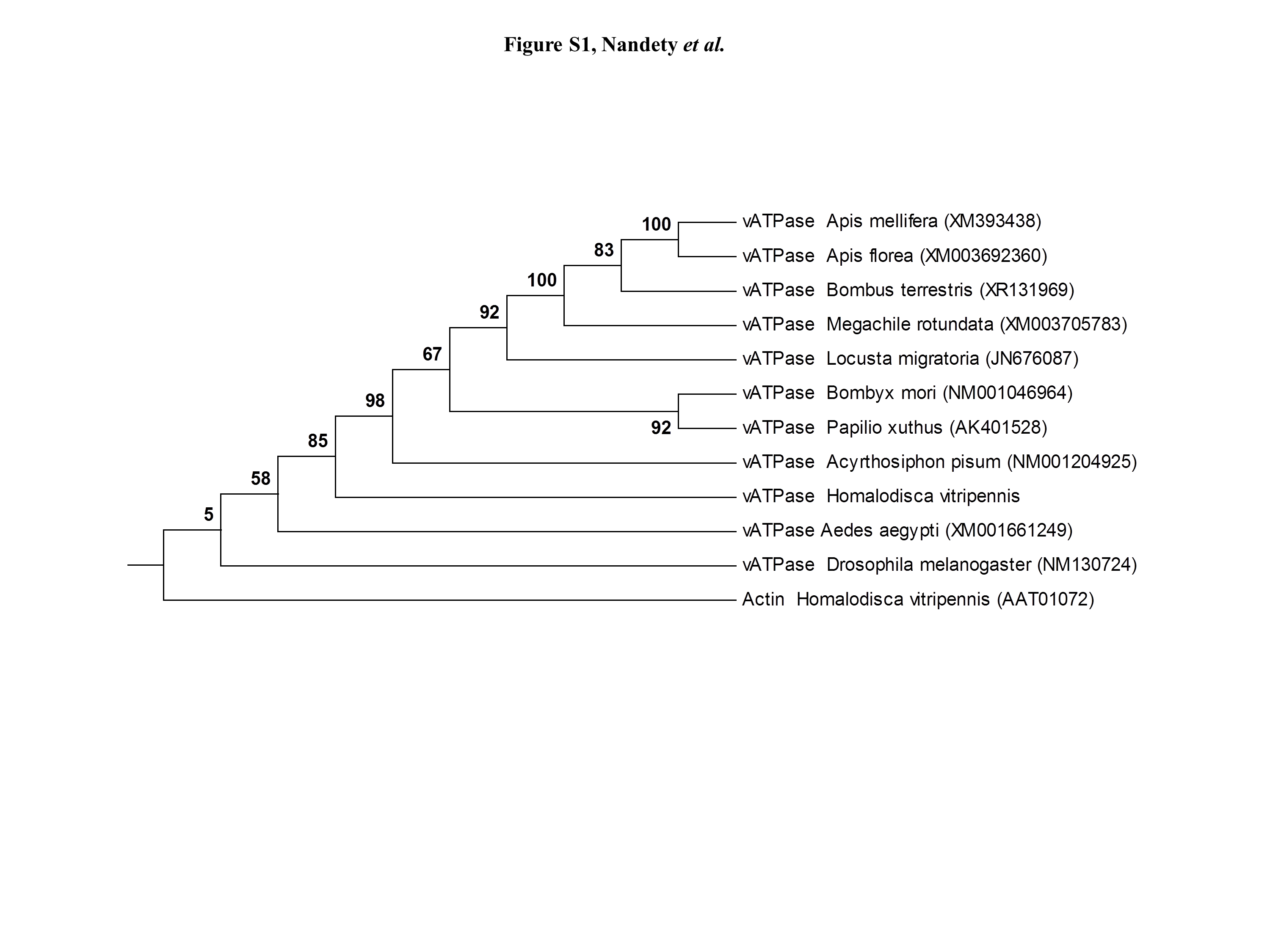

Supplement: Figure S1 — Phylogenetic tree of vacuolar ATPase encoded in the Homalodisca vitripennis transcriptome. The evolutionary relationship of H. vitripennis vacuolar ATPase with other insect vATPase proteins was shown. The evolutionary history was inferred using the Neighbor-Joining method. The percentage of replicate trees in which the associated taxa clustered together in the bootstrap test (500 replicates) is shown next to the branches. The evolutionary distances were computed using the Maximum Composite Likelihood method and are in the units of the number of base substitutions per site. All ambiguous positions were removed for each sequence pair. Evolutionary analyses were conducted in MEGA5. (TIF) [file pone.0081681.s001.tif]
