# Supplementary material for: Sequencing and De Novo Assembly of the Transcriptome of the Glassy-Winged Sharpshooter (Homalodisca vitripennis)
Source: PLoS One. 2013 Dec 10;8(12):e81681. doi: 10.1371/journal.pone.0081681 (PMC3858241; doi:10.1371/journal.pone.0081681)
Supplement: Table S1 — Primer pairs designed for the amplification of transcripts from Homalodisca vitripennis whole adults through the use of reverse transcriptase PCR. (DOCX) [file pone.0081681.s002.docx]

**Table S1**. Primer pairs designed for the amplification of transcripts from *Homalodisca vitripennis* whole adults through the use of reverse transcriptase PCR

| **No.** | **Primer Name** | **Sequence (5'-3')** |
| --- | --- | --- |
| 1 | *vATPase* for | CGTCCCGCACGCTCGTAGATGG |
| 2 | *vATPase* rev | CGTGTTGGTCATCCTGACCG |
| 3 | *ago2* for | CTTGCCGTCCTCATCCTCTCTCCT |
| 4 | *ago2* rev | TGATGGAAGCTGGGTTCAACCGAG |
| 5 | *actin* for | ATGTGTGACGACGACGTAGC |
| 6 | *actin* rev | GGGGAGCGATGATCTTGATC |
| 7 | *cuticle* for | CAGTGAAAGTCCCTGTGGATCG |
| 8 | *cuticle* rev | TTCACGGGTACGGGGATGTG |
| 9 | *chitin deacitilase* for | GCAGAAGGAGCATGACGTGG |
| 10 | *chitin deacitilase* rev | TTCTCGCCGTTGCAGAAAAG |
| 11 | *dicer* for | GCTTGCAGAAGGAAGCATCTGTCGT |
| 12 | *dicer* rev | TCAACAGGTCCACTTGTTGCAGGC |
| 13 | *jhamt* for | CGACAGAAGTGCTCCTTCAG |
| 14 | *jhamt rev* | CTCAATTCGGAACACAGGTGCC |
| 15 | *jheh* for | GGCTTGGTAGCTTGCAGATGC |
| 16 | *jheh* rev | GCCAACTACTGGAATGATGGCG |
| 17 | *jhe* for | CGAGGATAGTATTATTCCCGGC |
| 18 | *jhe* rev | GCAAAGGCACTCAATGCTGTAC |
| 19 | *serpin b6* for | TAGTCCCATCCGGACGACTCAGCCCTACTT |
| 20 | *serpin b6* rev | GGACCGGGACAAGCTCTAATGATTGTTCAA |
| 21 | *sugar transporter* for | CTAATGTCTGCACCAGCATGT |
| 22 | *sugar transporter* rev | CCTGTTCCTCCCAGAAAACA |
| 23 | *zinc metalloproteinase* for | CAGTTTACAGAGAAATGTGGGC |
| 24 | *zinc metalloproteinase* rev | GCACTCGTCTCCCAGGTTTA |
| 25 | *ubiquitin* for | CAAGACCCTGACTGGCAAGAC |
| 26 | *Ubiquitin* rev | GCCTCCACGAAGACGGAGAAC |
